# Identification and Characterization of a δ-Cadinol Synthase Potentially Involved in the Formation of Boreovibrins in *Boreostereum vibrans* of Basidiomycota

**DOI:** 10.1007/s13659-016-0096-4

**Published:** 2016-04-01

**Authors:** Hui Zhou, Yan-Long Yang, Jun Zeng, Ling Zhang, Zhi-Hui Ding, Ying Zeng

**Affiliations:** 1 State Key Laboratory of Phytochemistry and Plant Resources in West China, Kunming Institute of Botany, Chinese Academy of Sciences, 132 Lanhei Road, Kunming, 650201 China; 2 University of Chinese Academy of Sciences, Beijing, 100049 China

**Keywords:** Delta-cadinol, Sesquiterpene synthase, Biosynthesis, GC–MS, Fungi

## Abstract

**Abstract:**

Sesquiterpenoids are very common among natural products. A large number of sesquiterpene synthase genes have been cloned and functionally characterized. However, until now there is no report about the δ-cadinol synthase predominantly forming δ-cadinol (syn. torreyol) from farnesyl diphosphate. Sesquiterpenoids boreovibrins structurally similar to δ-cadinol were previously isolated from culture broths of the basidiomycete fungus *Boreostereum vibrans*. This led us to expect a corresponding gene coding for a δ-cadinol synthase that may be involved in the biosynthesis of boreovibrins in *B*. *vibrans*. Here we report the cloning and heterologous expression of a new sesquiterpene synthase gene from *B*. *vibrans*. The crude and purified recombinant enzymes, when incubating with farnesyl diphosphate as substrate, gave δ-cadinol as its principal product and thereby identified as a δ-cadinol synthase.

**Graphical Abstract:**

A new sesquiterpene synthase gene was cloned from the basidiomycete fungus *Boreostereum vibrans* and heterologously expressed in *E. coli*. The purified recombinant enzyme gave δ-cadinol as its principal product from farnesyl diphosphate and thereby identified as a δ-cadinol synthase (BvCS).
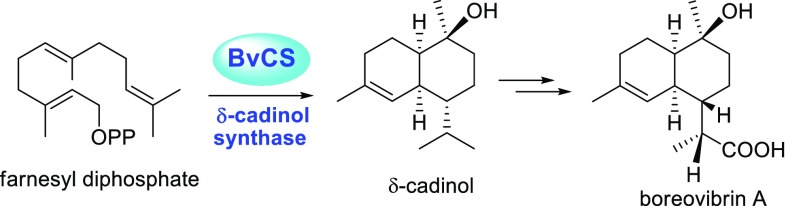

**Electronic supplementary material:**

The online version of this article (doi:10.1007/s13659-016-0096-4) contains supplementary material, which is available to authorized users.

## Introduction

**Fig. 1:**
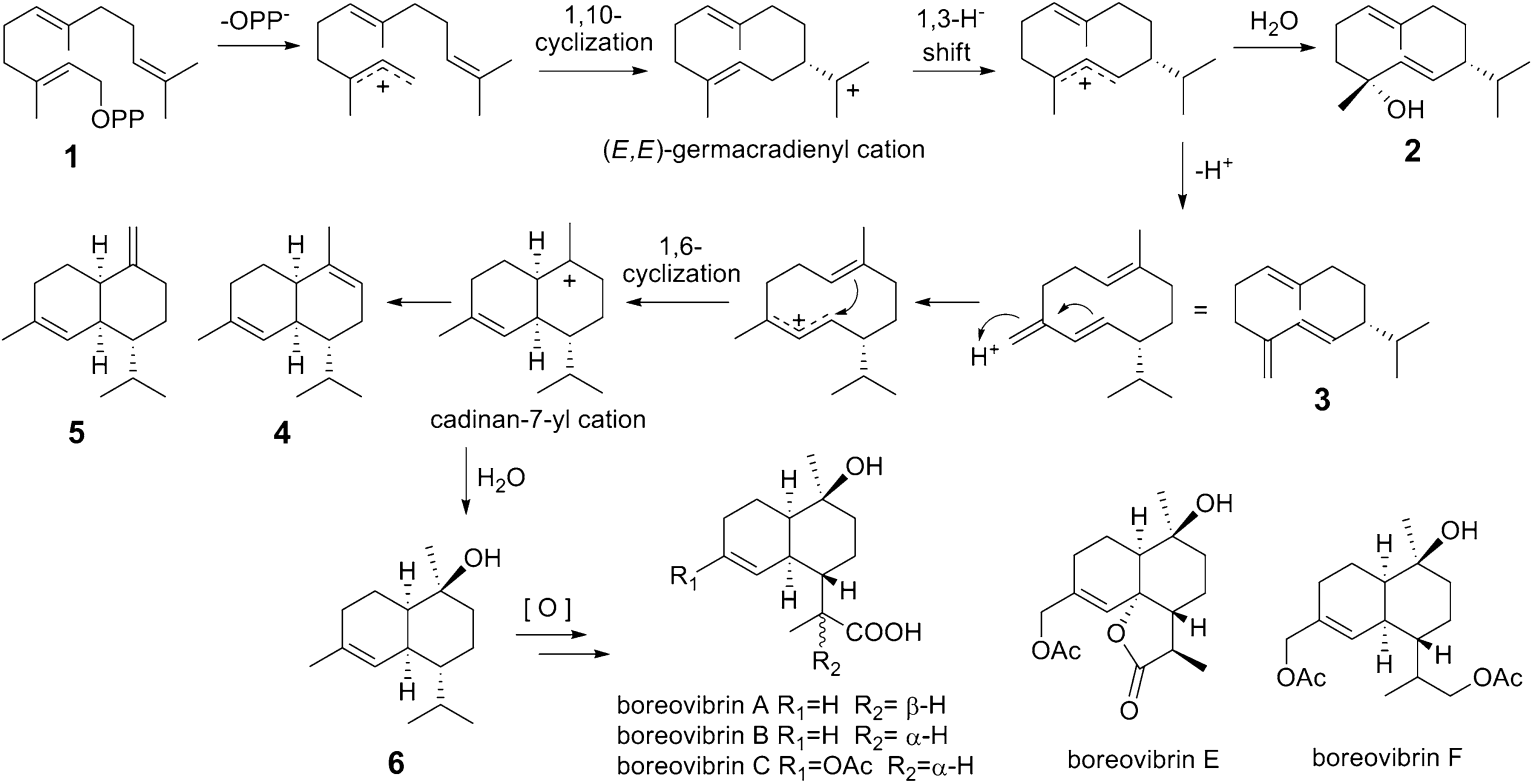
Proposed biosynthetic pathway to the sesquiterpenoids formed by the δ-cadinol synthase BvCS from *B. vibrans*

Higher fungi (Basidiomycota), among the many diverse organisms, are a major source of biologically active natural products, since they harbor a huge reservoir of active secondary metabolites [1–4]. From culture broths of the basidiomycete fungus *Boreostereum vibrans* (syn. *Stereum vibrans*), vibralactones with distinctly skeletons were identified [5–9] and generated by unusual and divergent biosynthetic pathways that we have recently established by precursor feedings in combination with genome mining [10, 11]. Further analyses of our *B. vibrans* genome draft assembly revealed several sequences to encode putative sesquiterpene synthases (STS). Interestingly, cadinane sesquiterpenoids boreovibrins A–G were isolated from *B. vibrans* [12]; most of them are structurally similar to δ-cadinol (syn. torreyol). Moreover, production of (+)-δ-cadinol was observed in mycelial of *Stereum hirsutum* which falls into the same genus with *B. vibrans* [13]. Thus we speculate that a sesquiterpene synthase catalyzing the conversion of farnesyl diphosphate (FPP, **1**) into δ-cadinol (**6**) is probably responsible for the production of boreovibrins (Fig. 1). Like other terpene synthases, STS catalyze the release of diphosphate from FPP and then guide migration of the reactive carbocation along the prenyl chain, thereby inducing a series of cyclization and rearrangement reactions, until final carbocation quenching by deprotonation or water [14]. The reaction with the nucleophile water can afford terpene alcohols as direct products instead of hydrocarbon terpenes. Many sesquiterpene alcohols have been detected in enzymatic products of STS from plants and bacteria, but major products similar to **6** are only found for τ-cadinol synthase (LaCADS) from the plant *Lavandula angustifolia* [15], and T-muurolol synthases from the bacteria *Streptomyces clavuligerus* [16] and *Roseiflexus castenholzii* [17, 18]. With **6** as a minor product, the sesquiterpene synthase Mg25 from the plant *Magnolia grandiflora* was shown to mainly produce β-cubebene from **1**, hence named as a β-cubebene synthase [19]. Even now there is no report about the δ-cadinol synthase predominantly forming δ-cadinol from FPP. Basidiomycete fungi are known to produce numerous bioactive sesquiterpenoid metabolites, yet only a few STS have been cloned and functionally characterized. For examples, the protoilludene synthase in *Armillaria gallica* was observed for exclusive production of Δ^6^-protoilludene and involved in the biosynthesis of melleolides [20]. Six STS from *Coprinus cinereus* were identified to produce germacrene A, α-muurolene (**4**), germacrene D (**3**), cubebol, and α-cuprenene as major products, respectively [21, 22]; nine STS from *Omphalotus olearius* to produce α-muurolene (**4**), β-elemene, δ-cadinene, γ-cadinene, Δ^6^-protoilludene, α-barbatene, *trans*-dauca-4(11),8-diene as major products, respectively [23]; and five STS from *Stereum hirsutum* to produce δ-cadinene, β-barbatene, Δ^6^-protoilludene as major products, respectively [24]. Here we report the cloning and heterologous expression of a new sesquiterpene synthase gene from *B*. *vibrans*. The purified recombinant enzyme, when incubating with **1** as a substrate, gave δ-cadinol as its principal product and thereby identified as a δ-cadinol synthase.

## Results and Discussion

Based on the putative sesquiterpene synthase sequence from *B. vibrans* genome draft assembly, the full-length cDNA (designated as *BvCS*) was recovered by RT-PCR with specific primers and contained an open reading frame of 1182 nucleotides. The sequence has been submitted to the GenBank database under accession number KU668561. The deduced amino acid sequence of *BvCS* had 49 % identity to α-muurolene synthase (Cop3, accession no. A8NE23) from *Coprinus cinereus* [21] and germacradienol/germacrene D synthase (accession no. KNZ73785) from *Termitomyces* sp. J132. Showing 22 % identity, BvCS was less related to δ-cadinene synthase (accession no. XP_007299839) from *Stereum hirsutum* [24] (Table S1 in Electronic supplementary material).

**Fig. 2:**
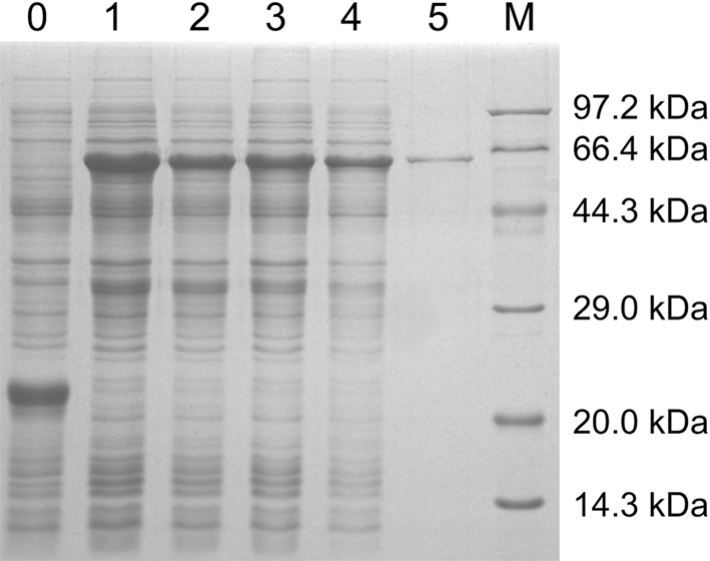
Expression of *BvCS*/pET32a+ in *E. coli* and purification of recombinant fusion proteins. *0* Empty vector; *1* whole proteins; *2* soluble proteins; *3* unbinding proteins; *4* washing; *5* elute (the purified enzyme); *M* protein size marker

**Fig. 3:**
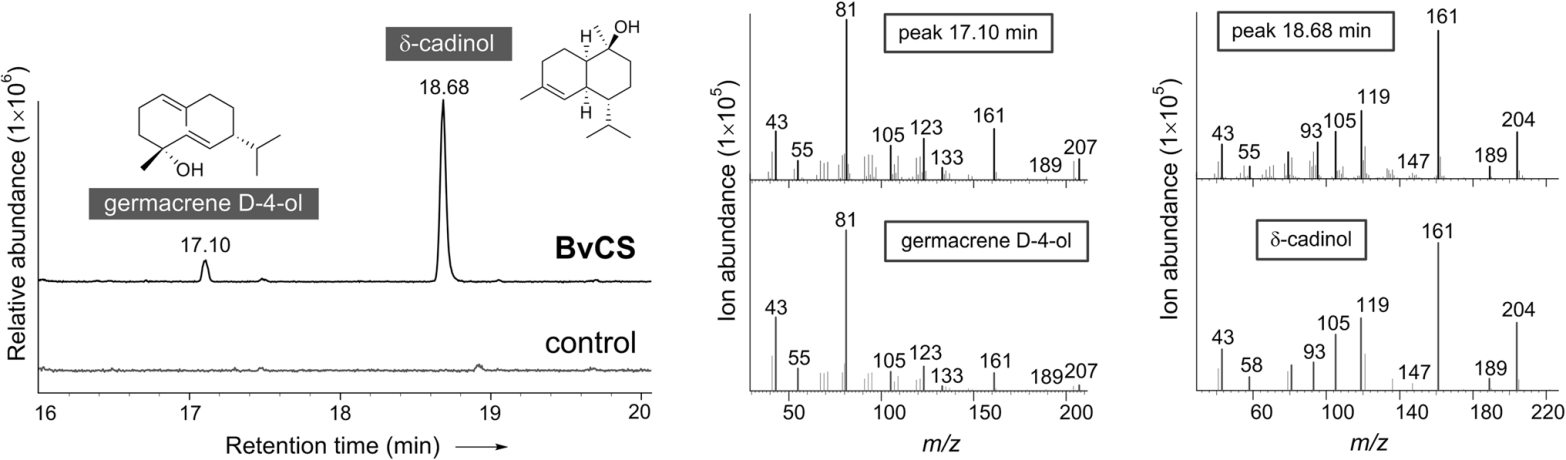
GC–MS analyses of products formed by the recombinant BvCS and the heat-denatured enzyme as control, respectively, with FPP as substrate. Total ion chromatograms (*left*) and the corresponding mass spectra (*right*) illustrate the product peaks at 17.10 and 18.68 min matching germacrene D-4-ol and δ-cadinol, respectively, in mass fragmentation patterns included in the database

Next, functional expression in pET32a+/*Escherichia coli* BL21(DE3) was conducted to confirm the catalytic activity of BvCS. Soluble protein expression was achieved at 15 °C for 22 h with 0.1 mM IPTG (isopropyl-β-d-thiogalactopyranoside), as determined by SDS-PAGE analysis (Figure S1 in Electronic supplementary material). The crude enzyme was assayed for sesquiterpene synthase activity using **1** as a substrate under optional condition as described in Sect. 3. Based on GC–MS analyses, major product peak at 18.68 min (retention time) and minor products were observed for crude BvCS, compared with the empty vector as control (Electronic supplementary material). The purified recombinant protein was used for further characterization. After purification under native condition on the Ni–NTA Agarose, the analysis of the elute on SDS-PAGE led to detection of the main band corresponding exactly to the predicted size of the recombinant BvCS protein of approximately 61 kDa (Fig. 2). When incubated with FPP, the purified BvCS made δ-cadinol (**6**) as major product at 18.68 min, and minor products including germacrene D-4-ol (**2**) at 17.10 min, α-muurolene (**4**) at 15.32 min, and γ-muurolene (**5**) at 14.76 min, compared with the heat-denatured enzyme as control (Fig. 3, Electronic supplementary material). This is detected and characterized by GC–MS following comparison to the standards included in the database. The product peak at 18.68 min generated the dominant mass segments at *m/z* 161, 119, 204 and 105 perfectly matching δ-cadinol (also torreyol) in mass spectra of the database and the authentic δ-cadinol in publications [13, 25] (Electronic supplementary material).

For the biosynthesis of cadalane sesquiterpenes, three alternative mechanisms were proposed and elucidated in bacteria [16, 17] and plants [25, 26]. The intermediacy of nerolidyl diphosphate (NPP) was generally accepted. Recently, however, the pathway via the protonation of the neutral intermediate **3** was demonstrated through labeling experiments to account for production of 92 % of cadalane sesquiterpenes by MtTPS5 from the plant *Medicago truncatulathe*, which can give **6** from **1** as its minor product [25]. Furthermore, the germacrene D (**3**) pathway was proposed for the bacterial STS from *Chitinophaga pinensis* DSM 2588 that can afford both δ-cadinene and germacrene D-4-ol (**2**), although no germacrene D was present in the enzymatic products [17]. As shown in this study, BvCS can form **6** as major product and **2** as minor product (Fig. 3). Therefore it is likely for the enzyme to follow the germacrene D pathway as that from *C. pinensis* DSM 2588 [17] (Fig. 1, Electronic supplementary material), while alternative mechanisms with the intermediacy of NPP could also be possible.

In conclusion, we cloned the full-length cDNA of a new sesquiterpene synthase gene from the basidiomycete *Boreostereum vibrans* and expressed it in *E. coli* for functional characterization. Based on GC–MS analyses, the recombinant enzyme was demonstrated to mainly produce δ-cadinol from farnesyl diphosphate and thereby identified as a δ-cadinol synthase.

## Experimental Section

### Gene Cloning

Mycelia of the fungus *Boreostereum vibrans* was inoculated in 0.5 L modified PDB medium (potato 200.0 g, glucose 20.0 g, KH_2_PO_4_ 3.0 g, MgSO_4_ 1.5 g, citric acid 0.1 g, and thiamin hydrochloride 10 mg in 1 L of deionized water, pH 6.5), cultured at 25 °C on a rotary shaker at 140 rpm. Mycelia were activated on PDB agar plates before inoculation in liquid PDB. Total RNA was isolated from the mycelia on day 20 using the Plant RNA Mini Kit (Qiagen). The first-strand cDNA was synthesized with Superscript™ III First-strand Synthesis System (Invitrogen). The full length cDNA was obtained with specific primer pairs 5′-CCCGACCTTCTCACCATCTGT-3′ (forward) and 5′-CGCGAGGTATAGAGCACCTGT-3′ (reverse) according to the predicted gene sequence in our *B. vibrans* genome draft assembly. For PCR, 30 cycles of reactions were performed at the condition (95 °C, 30 s; 58 °C, 30 s; 72 °C, 100 s) with final extension at 72 °C, 10 min. The amplicons were cloned and sequenced to verify the encoding region. The sequence, designated as *BvCS*, has been submitted to the GenBank database under accession number KU668561.

### Expression in *E. coli* and Enzyme Purification

The ORF of *BvCS* was cloned into the expression vector pET32a+ (Novagen) which was subsequently transformed into *E. coli* BL21(DE3) (Novagen) for a fusion expression, using the original pET32a+ as negative control. Protein expression at 37 or 15 °C with 0.1 or 0.5 mM IPTG was determined by SDS-PAGE analysis. Cells induced with 0.1 mM IPTG at 15 °C for 22 h were collected by centrifugation (8000 rpm, 4 °C, 5 min), washed by deionized water and suspended in 50 mM Tris–HCl buffer (pH 7.5) for crude enzyme preparation or in the binding buffer (20 mM Tris–HCl pH 7.5, 0.3 M NaCl, 5 mM imidazole) for enzyme purification. The cell lysate obtained by sonication on ice was then centrifuged at 12000 rpm for 10 min at 4 °C and the supernatant, containing the soluble recombinant enzyme was used for crude enzyme assay or enzyme purification. Purification of His-tagged enzymes was performed according to the Ni–NTA Agarose protocol (Qiagen) with a wash buffer (20 mM Tris–HCl pH 7.5, 0.5 M NaCl, 150 mM imidazole) and an elution buffer (20 mM Tris–HCl pH 7.5, 0.5 M NaCl, 300 mM imidazole). The elute containing the purified enzyme was immediately desalted with 50 mM Tris–HCl buffer (pH 7.5), concentrated by centrifugation at 4 °C (Amicon Ultra-15, Merck Millipore), and immediately used for activity assays. All fractions were analyzed by SDS-PAGE on 12 % polyacrylamide gel under non-reduced condition at 140 V for 1.5 h.

### Enzyme Assays and GC–MS Analyses

Crude enzyme was assayed for sesquiterpene synthase activity using FPP (Sigma) of 10 μg in assay buffer (50 mM Tris–HCl pH 7.5, 10 % glycerol, 10 mM MgCl_2_, 1 mM dithiothreitol, 100 mM NaCl), incubating for 4 h at 30 °C, using the empty vector as control. After extraction with 2 mL hexane, the hexane phase was collected by centrifugation, dehydrated over anhydrous sodium sulfate and concentrated under a stream of air for GC–MS analysis. The purified enzyme activity was detected as described above with FPP of 5 μg, using heat-denatured enzyme as control.

GC–MS [Agilent HP6890/5973, column: 0.25 mm × 30 mm, 0.25 µm (HP-5MS)] was conducted under electron-impact (EI) mode (70 eV). The flow rate of helium carrier gas was set at 1.0 mL/min. Samples (2 μL) were injected at 80 °C. After holding the samples for 5 min at 80 °C, the column temperature was increased at 5 °C/min to 280 °C and hold for 30 min. The MS date was collected from 35 to 500 *m/z*. The identification of the compounds was achieved by comparing the retention time and the mass spectra with those of the standards included in the library (wiley7n.1) and the authentic compounds in publications.

## Electronic supplementary material

Below is the link to the electronic supplementary material.

Supplementary material 1 (PDF 1843 kb)
