# Supplementary material for: Identification and Characterization of a δ-Cadinol Synthase Potentially Involved in the Formation of Boreovibrins in Boreostereum vibrans of Basidiomycota
Source: Nat Prod Bioprospect. 2016 Apr 1;6(3):167–71. doi: 10.1007/s13659-016-0096-4 (PMC5385660; doi:10.1007/s13659-016-0096-4)
Supplement: Supplementary file 1 — Supplementary material 1 (PDF 1843 kb) [file 13659_2016_96_MOESM1_ESM.pdf]

Supplementary Material

Three alternative mechanisms for the biosynthesis of cadalane sesquiterpenes as presented in the publication of P. Rabe, J. S. Dickschat, *Angew. Chem. Int. Ed.* **52**, 1810–1812 (2013)

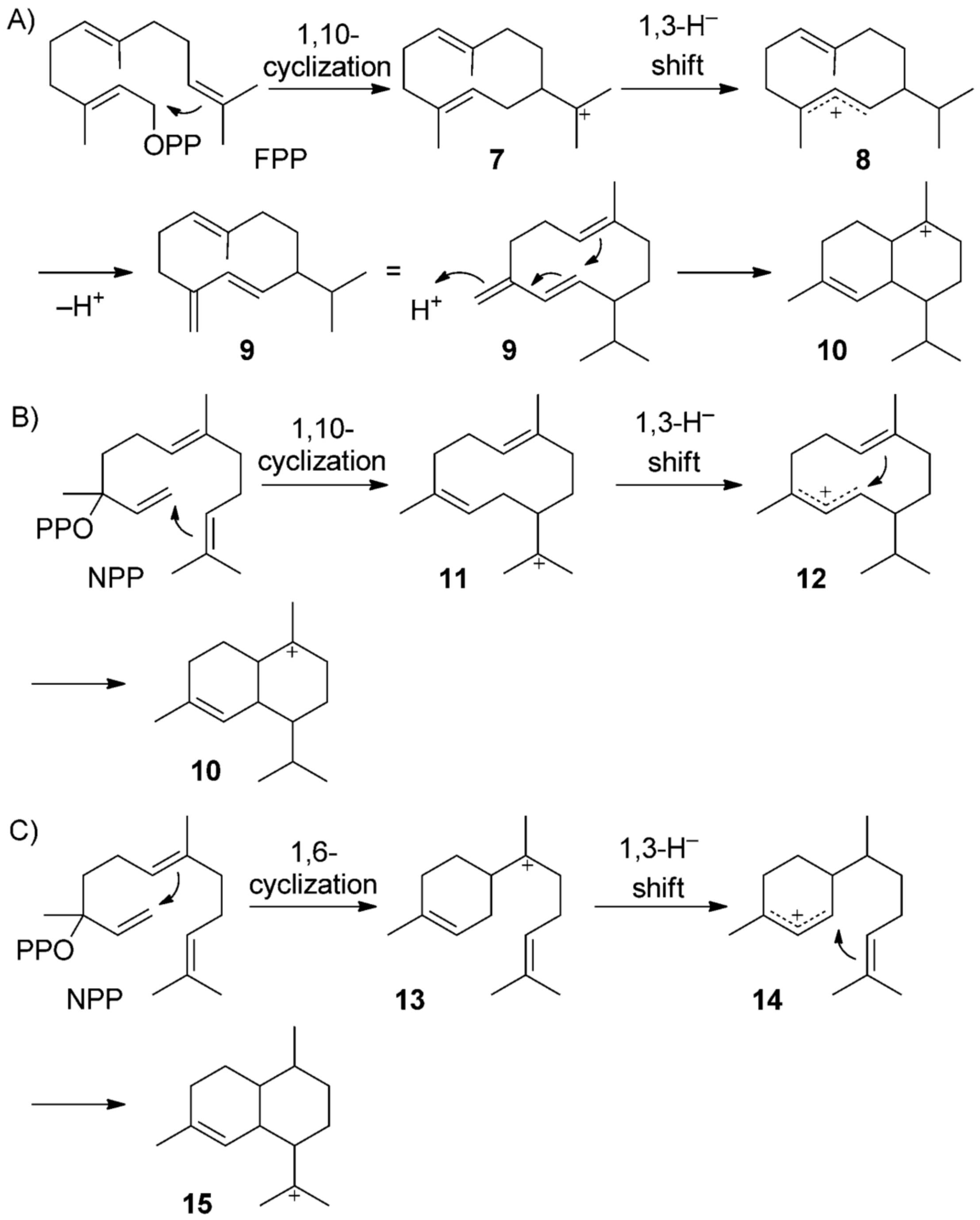

Table S1. Sequence identities (%) of the fungal homologs of Basidiomycota

	B. vibrans	*C. cinereus*	*Termitomyces* sp. J132	*S. hirsutum*
BvCS / *B. vibrans*	ID	0.492	0.489	0.225
α-muurolene synthase *Coprinus cinereus*		ID	0.559	0.228
germacradienol synthase *Termitomyces* sp. J132			ID	0.286
δ-cadinene synthase *Stereum hirsutum*				ID

Figure S1. Expression of *BvCS* in pET32a+ / *E. coli* BL21(DE3) detected by SDS-PAGE

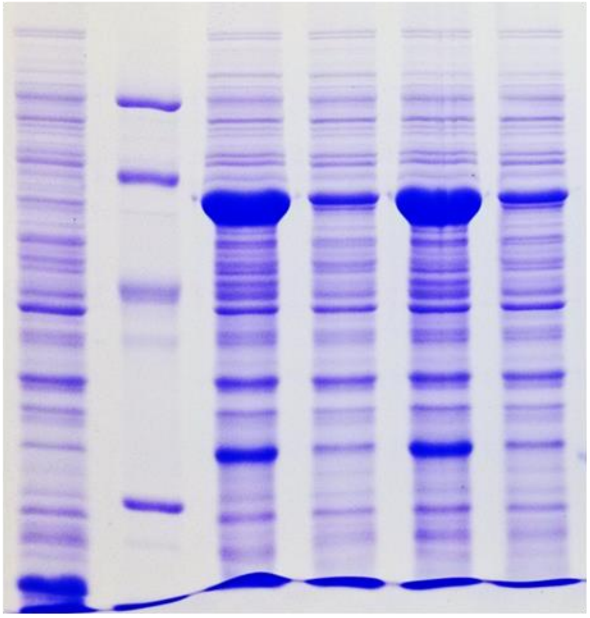

BvCS /pET32a+ in *E. coli* induced at 37°C for 8 h with 0.5 or 0.1 (right) mM IPTG

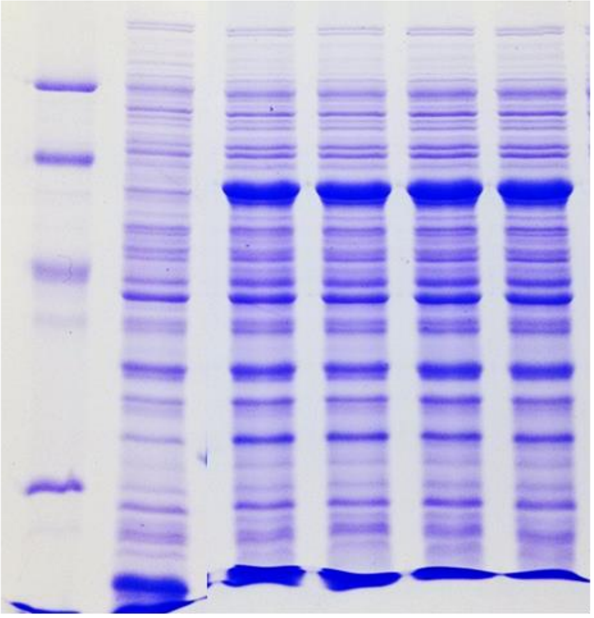

BvCS /pET32a+ in *E. coli* induced at 15°C for 22 h with 0.5 or 0.1 (right) mM IPTG

GC-MS analyses of **crude recombinant BvCS**, showing major product at 18.68 min and minor product at 17.10 min. Peak at 20.25 min is farnesol hydrolyzed from FPP as substrate by *E. coli* phosphatase.

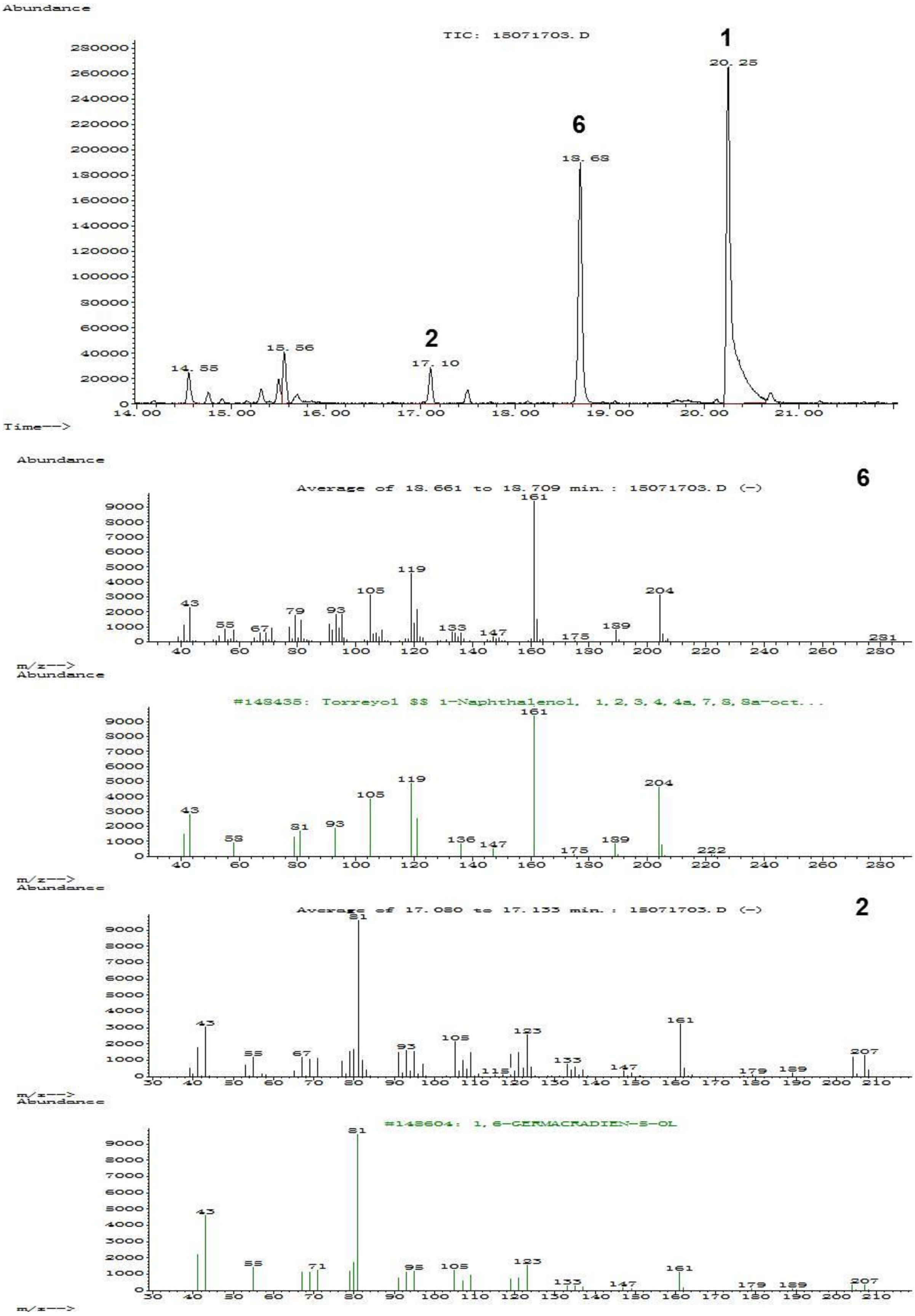

1

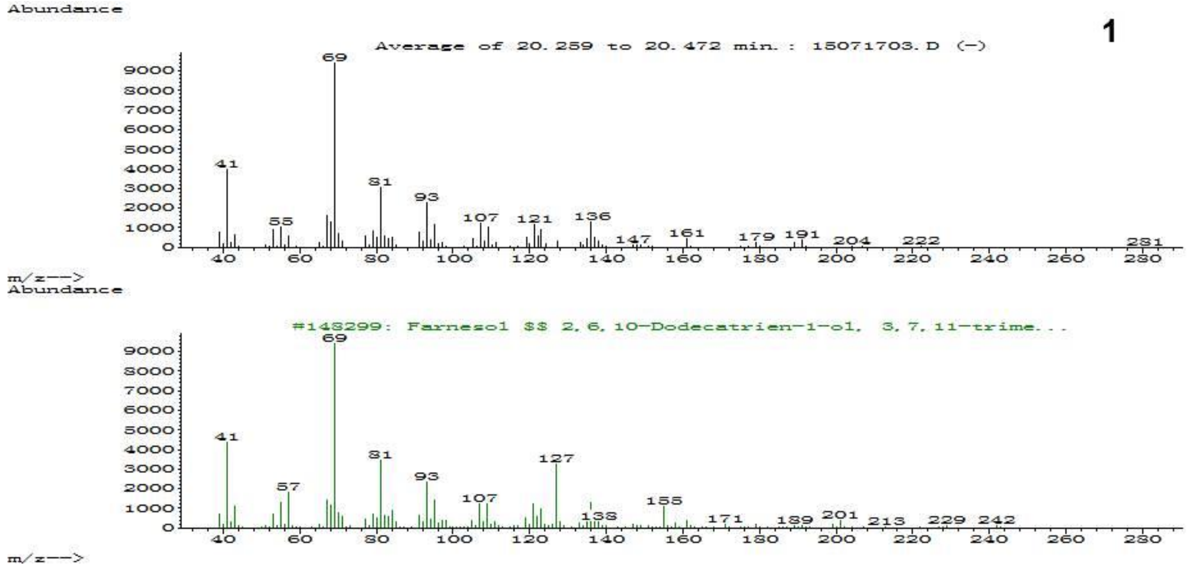

Empty vector as control

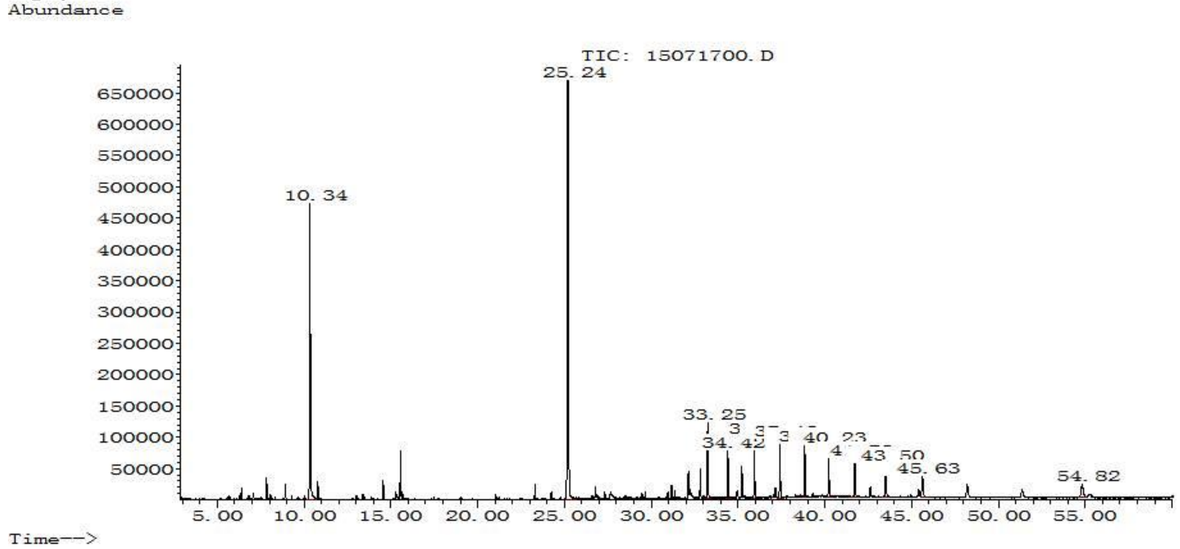

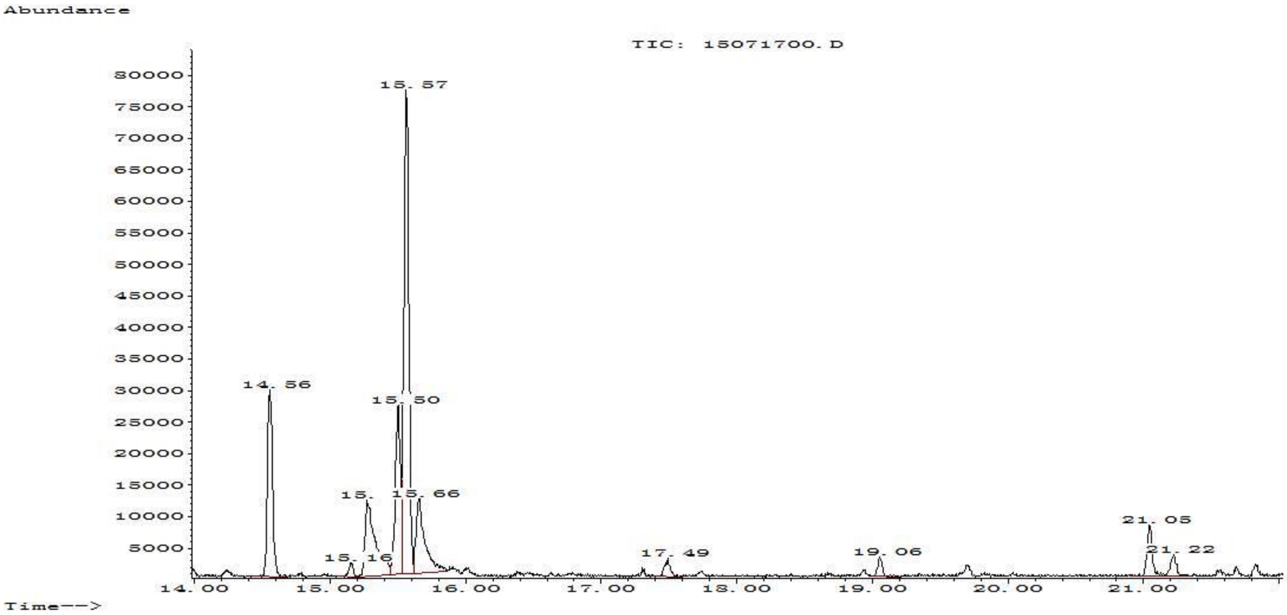

GC-MS analyses of the **purified recombinant BvCS**, showing major product at 18.68 min and minor products at 17.10 min, 15.32 min and 14.76min, compared to the heat-denatured BvCS as control.

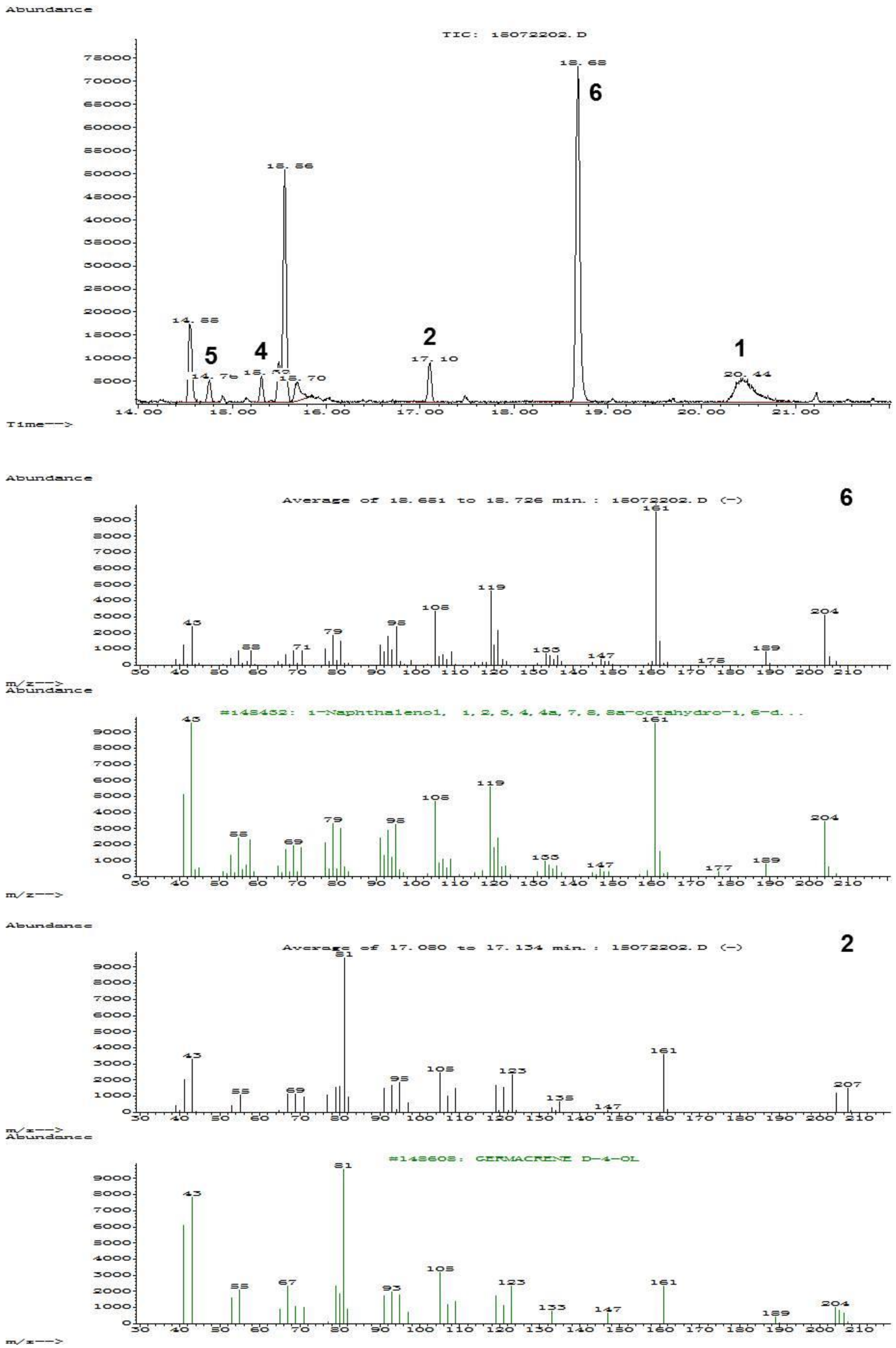

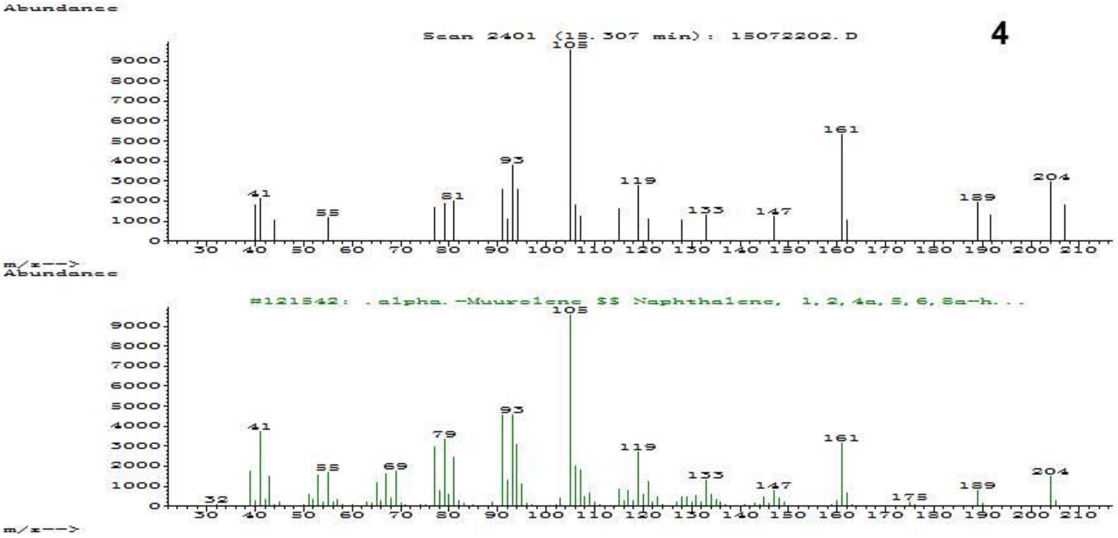
4
Abundance
Scan 2401 (15.307 min): 15072202.D
m/z-->
#121542: .alpha.-Muurolene $$ Naphthalene, 1,2,4a,5,6,8a-h...

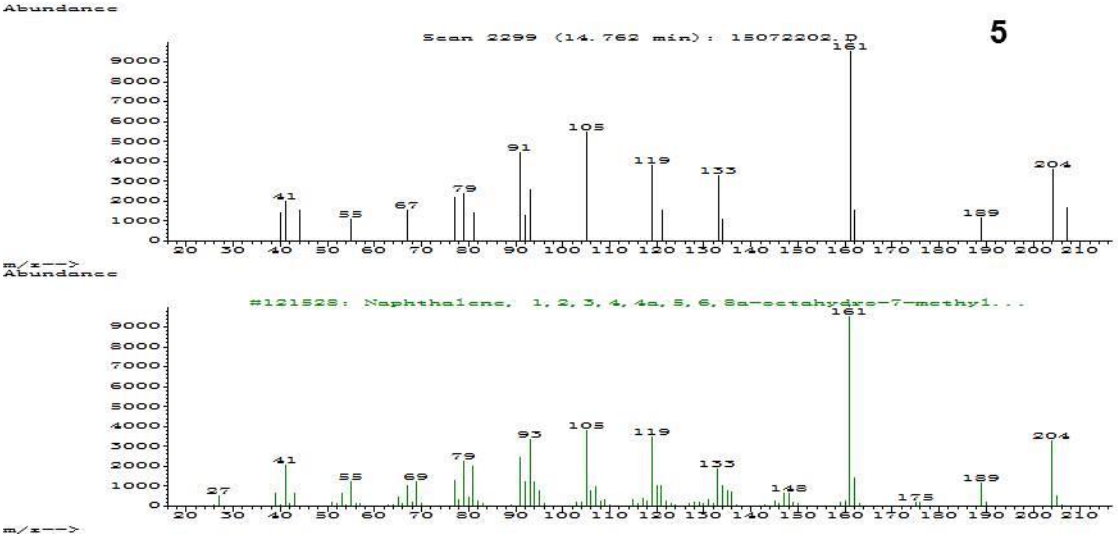
5
Abundance
Scan 2299 (14.762 min): 15072202.D
m/z-->
#121528: Naphthalene, 1,2,3,4,4a,5,6,8a-octahydro-7-methyl...

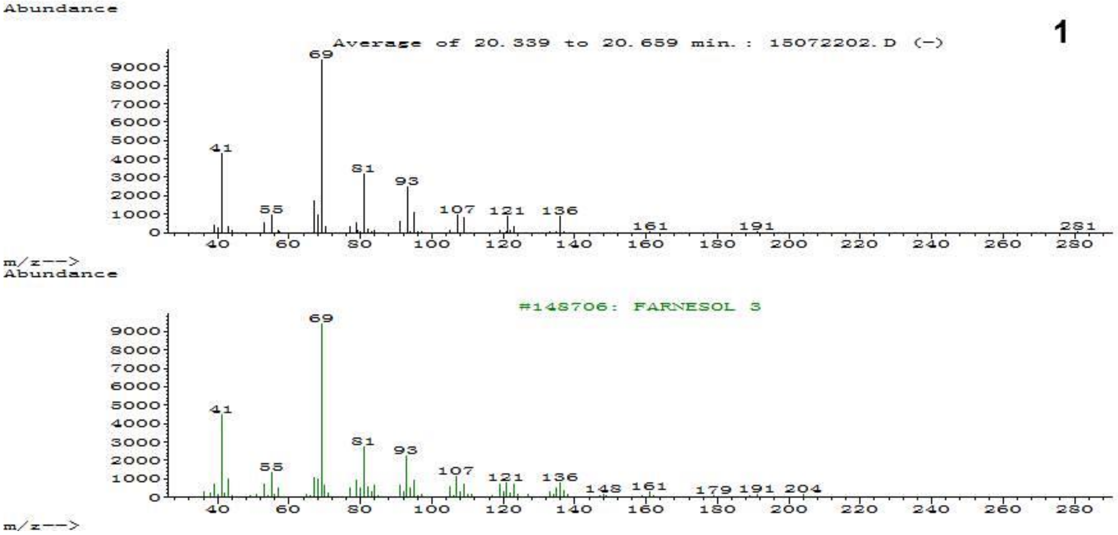
1
Abundance
Average of 20.339 to 20.659 min.: 15072202.D (-)
m/z-->
#148706: FARNESOL 3

GC-MS analyses of **heat-denatured BvCS as control**

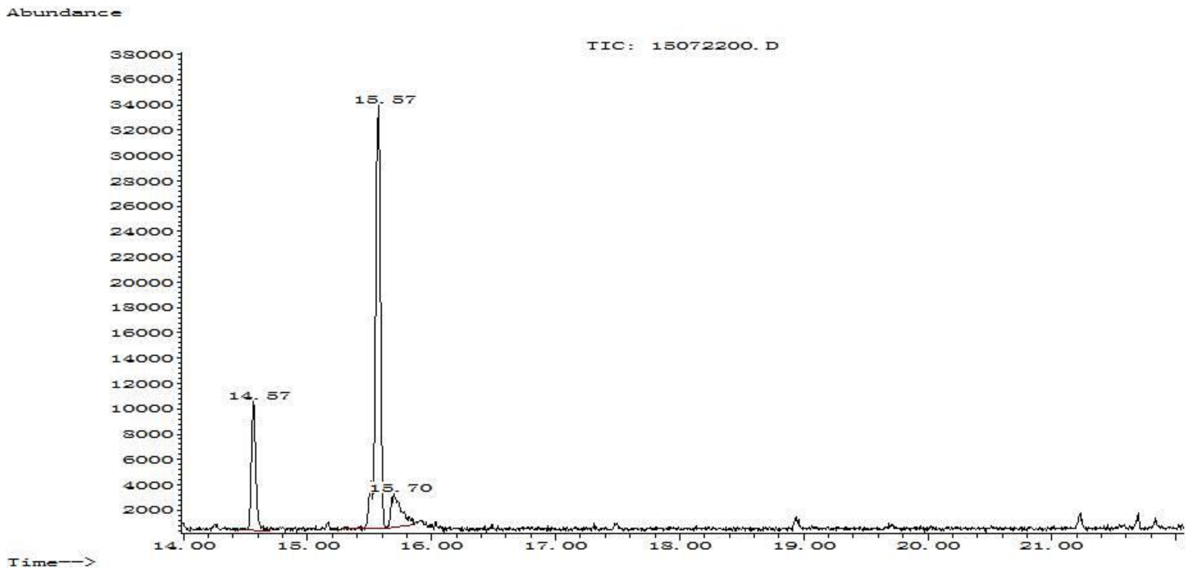

Mass spectra of the authentic δ-cadinol (syn. torreyol), γ-muurolene and α-muurolene in publications

1. Stefan Garms, Tobias G. K€ollner, and Wilhelm Boland. A Multiproduct Terpene Synthase from *Medicago truncatula* Generates Cadalane Sesquiterpenes via Two Different Mechanisms. *J. Org. Chem.* 2010, **75**, 5590–5600.

Mass spectrum of the authentic **(–)-torreyol** (reference) on Page 27 of the Supporting Information

torreyol (**31**)

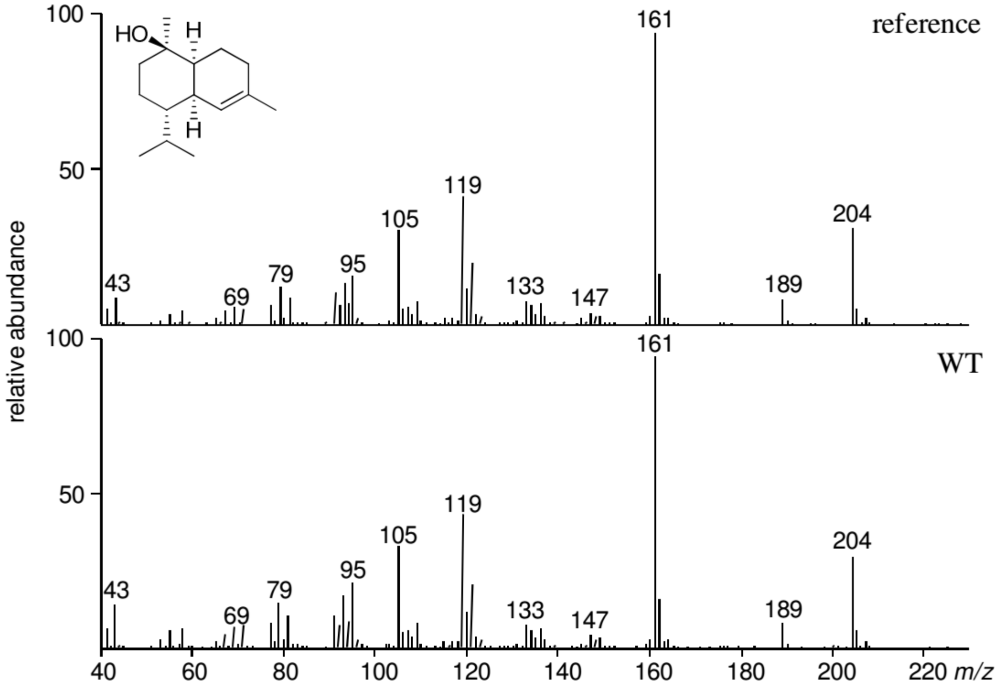

γ-muurolene (**16**)

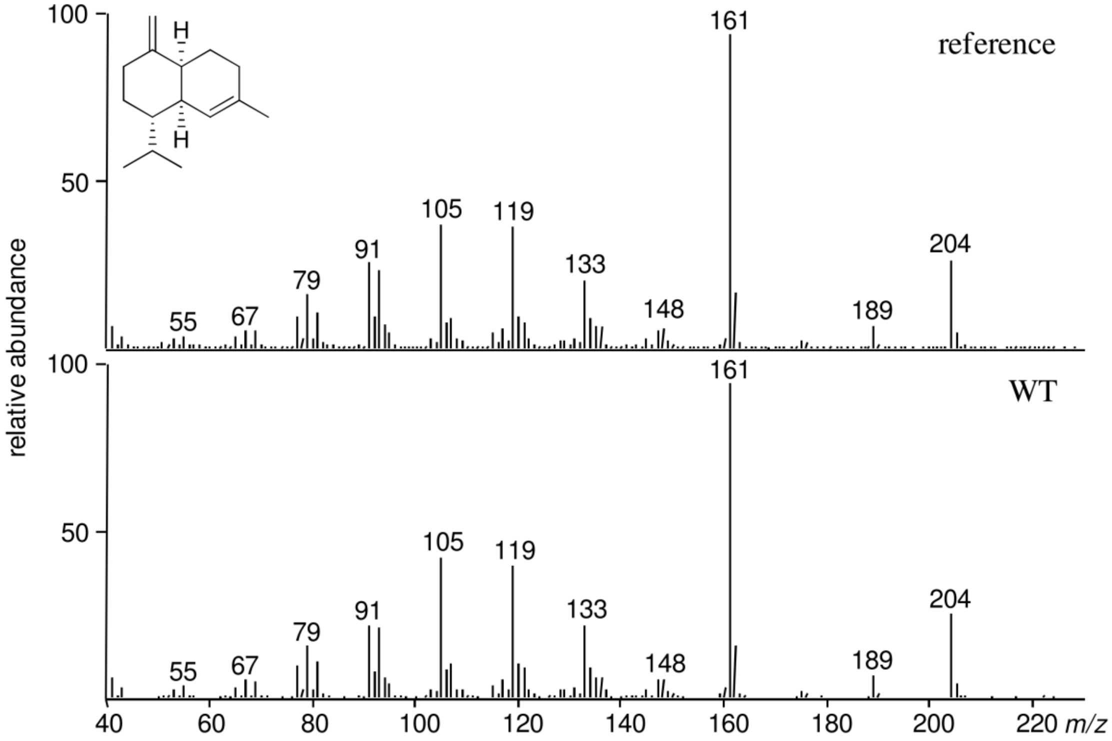

α-muurolene (**19**)

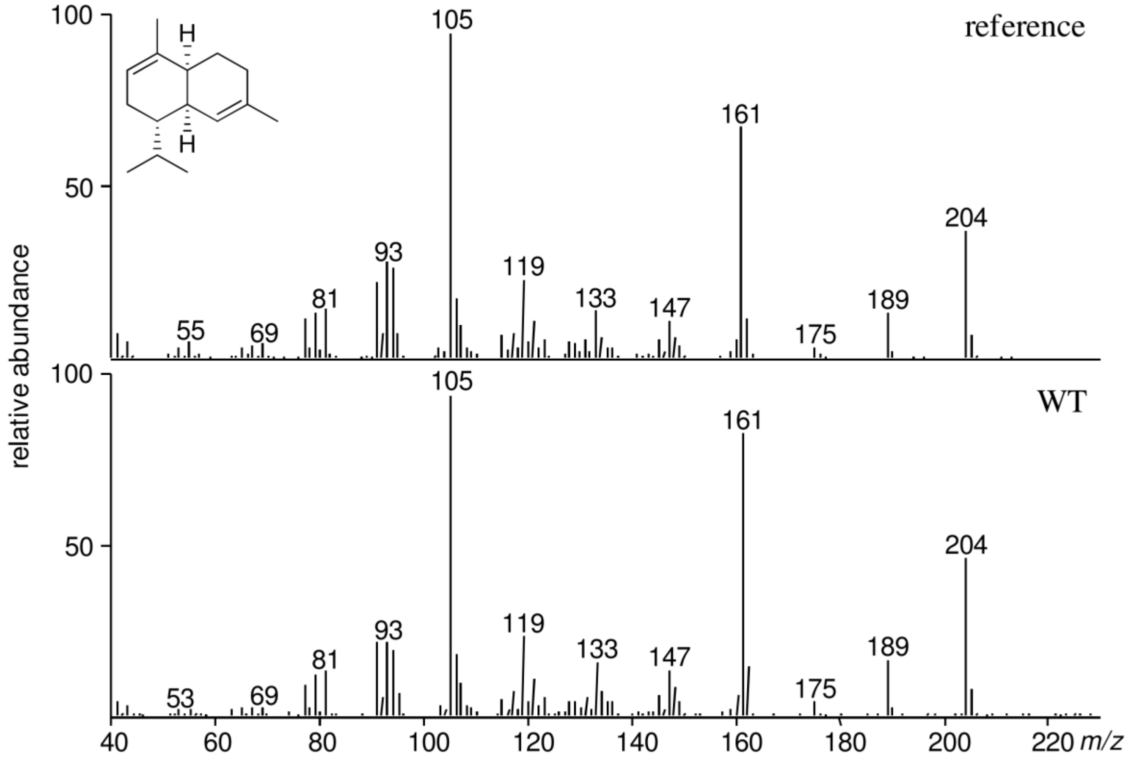

2. A. M. Ainsworth, A. D. M. Rayner, S. J. Broxholme, J. R. Beeching, J. A. Pryke, P. R. Scard, J. Berriman, K. A. Powell, A. J. Floyd, S. K. Branch. Production and properties of the sesquiterpene, (+)-torreyol, in degenerative mycelial interactions between strains of *Stereum*. *Mycol. Res.* 1990, **94** (6), 799–809.

Structural formula of (+)-torreyol.

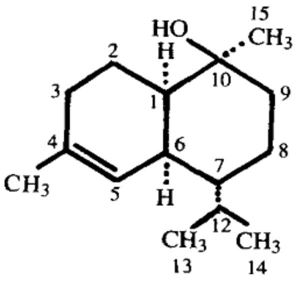

Mass spectrum: m/z (R.I. %) 204 (38), 189 (10), 161 (100), 119 (50), 105 (38), 95 (40)

Considerations for the germacrene D pathway

1. In the Supporting Information of the paper by P. Rabe & J. S. Dickschat (*Angew. Chem. Int. Ed.* 2013, **52**, 1810–1812), the germacrene D pathway (***Table 2*** and ***Scheme 2***) was proposed for the enzyme from *Chitinophaga pinensis* DSM 2588 producing both δ-cadinene and germacrene D-4-ol, although the putative intermediate germacrene D was not detected in the products. When the enzyme from *Roseiflexus castenholzii* DSM 13941 produced δ-cadinene and its similar products, the NPP pathway was proposed (***Table 6*** and ***Scheme 6***).

Table 2. Terpenes identified in headspace extracts of *E. coli* BL21 during heterologous expression of the γ-cadinene synthase from *Chitinophaga pinensis* DSM 2588 (YP_003124367).

Compound	*I*[a]	*I* (Lit.)	1. / %[b]	2. / %[b]
α-Copaene (**2a**)	1376	1374[1]	0.5	0.4
β-Elemene (**2b**)	1391	1389[1]	2.5	0.9
γ-Elemene (**2c**)	1434	1434[1]	0.3	0.2
(*E*)-β-Farnesene (**2d**)	1454	1454[1]	0.5	0.4
cis-Muurola-4(15),5-diene (**2e**)	1464	1465[1]	0.7	0.0
10-*epi*-Zonarene (**2f**)	1499	1501[1]	1.5	0.0
α-Muurolene (**2g**)	1501	1500[1]	3.0	2.1
γ-Cadinene (**2**)	1515	1513[1]	84.2	92.6
δ-Cadinene (**2h**)	1525	1522[1]	2.2	2.0
α-Cadinene (**2i**)	1539	1537[1]	1.3	1.5
Germacrene D-4-ol (**2j**)	1577	1574[1]	3.3	0.0

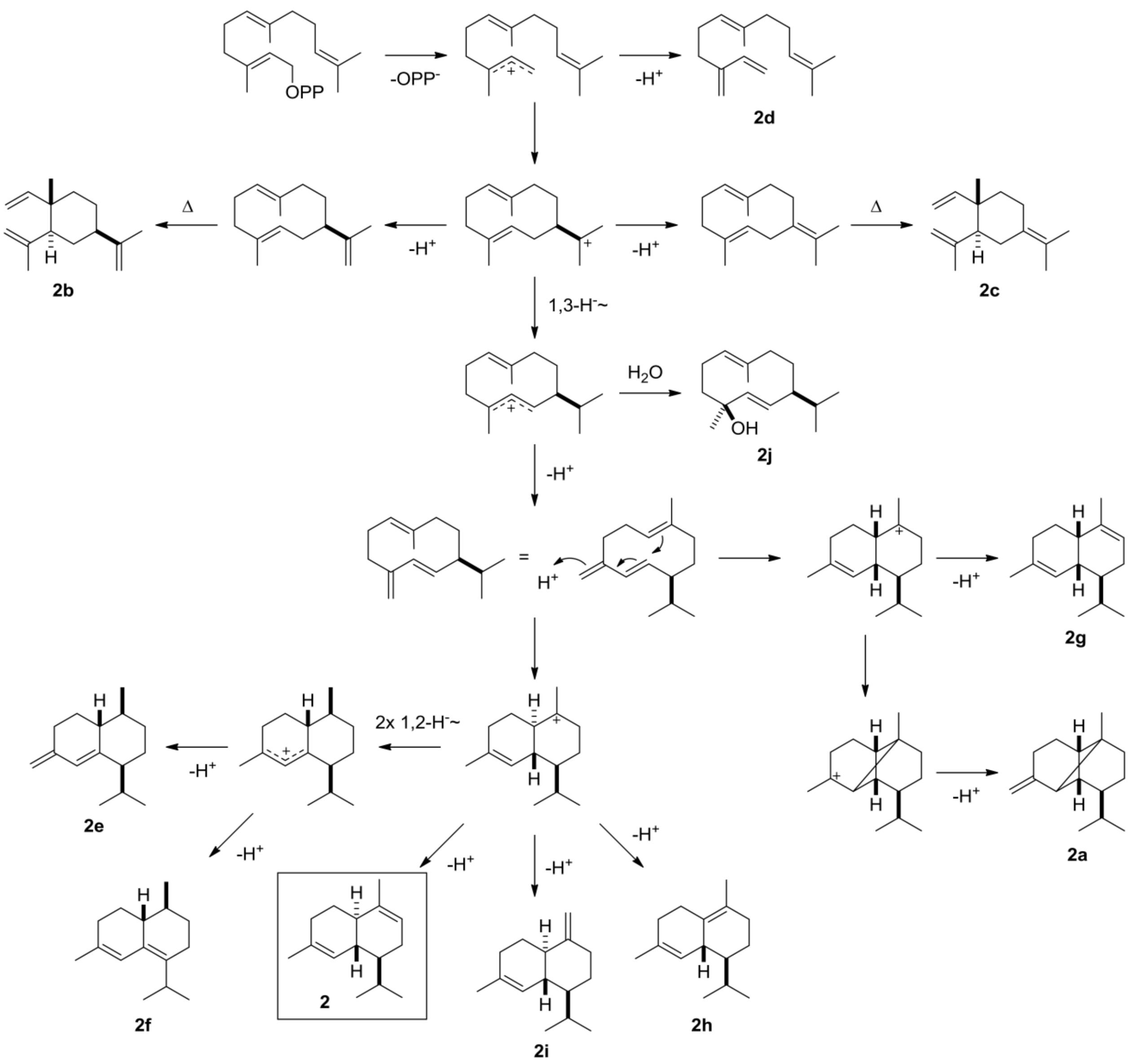

Scheme 2. Biosynthetic pathway to the sesquiterpenes produced by the γ-cadinene synthase from *C. pinensis* DSM 2588.

Table 6. Terpenes identified in headspace extracts of *E. coli* BL21 during heterologous expression of the T-muurolol synthase from *Roseiflexus castenholzii* DSM 13941 (YP_001430766).

**Compound**	***I*[a]**	***I* (Lit.)**	**1. / %[b]**	**2. / %[b]**
γ-Muurolene (**6a**)	1478	1478[1]	3.4	10.0
α-Muurolene (**6b**)	1501	1500[1]	5.0	14.7
δ-Cadinene (**6c**)	1523	1522[1]	1.9	15.6
T-Muurolol (**6**)	1640	1640[1]	89.7	59.6

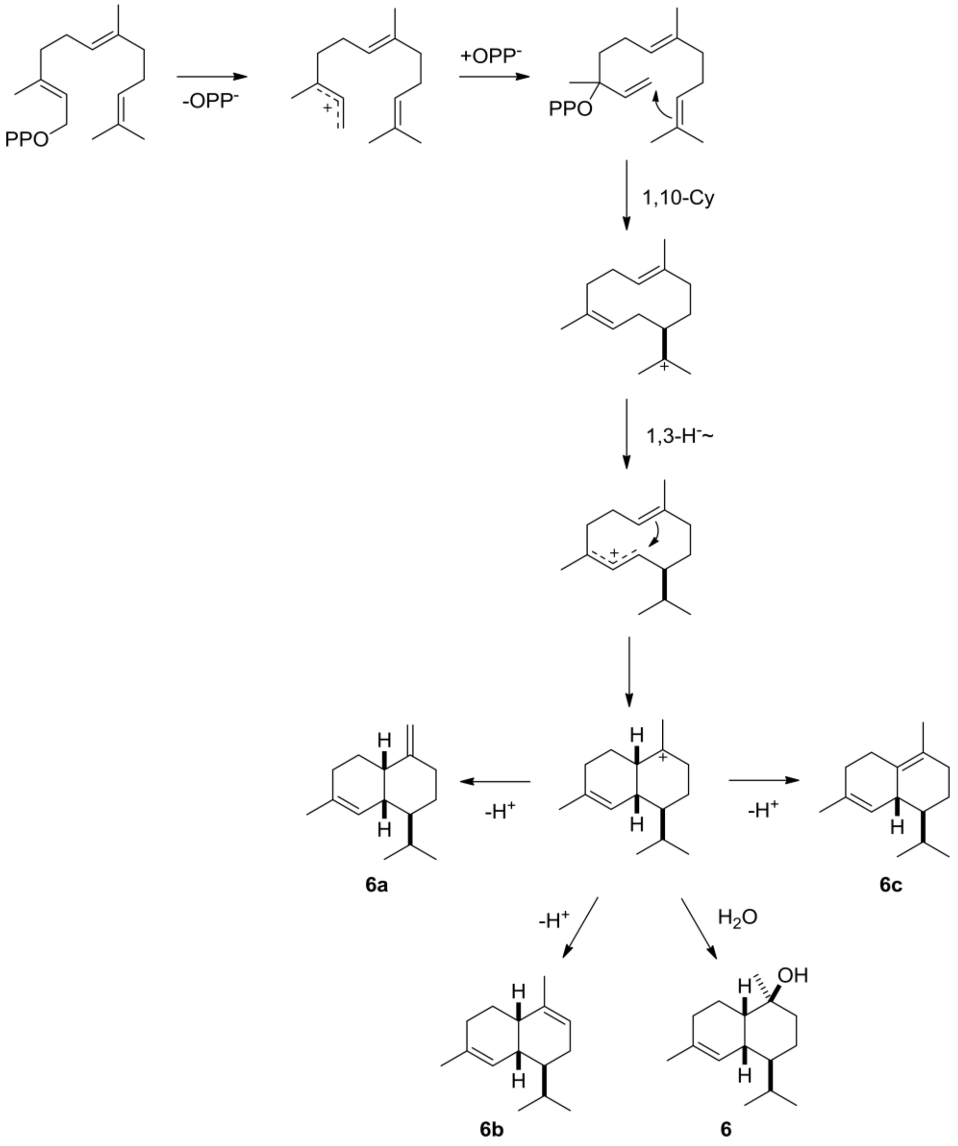

Scheme 6. Biosynthetic pathway to the sesquiterpenes produced by the T-muurolol synthase from *R. castenholzii* DSM 13941.

2. Garms et al (*J. Org. Chem.* 2010, **75**, 5590–5600) demonstrated that MtTPS5 can give torreyol (also **δ-cadinol**) as its minor product. Based on the labeling experiments conducted with deuterium oxide, cadalane sesquiterpenes are mainly (**92%**) produced via the protonation of the neutral intermediate germacrene D (highlighted **blue** in Scheme 6), and about **8%** arises from NPP pathway (highlighted **orange** in Scheme 6).

SCHEME 6. **Proposed Reaction Mechanism for the Formation of Sesquiterpene Products by MtTPS5**[a]

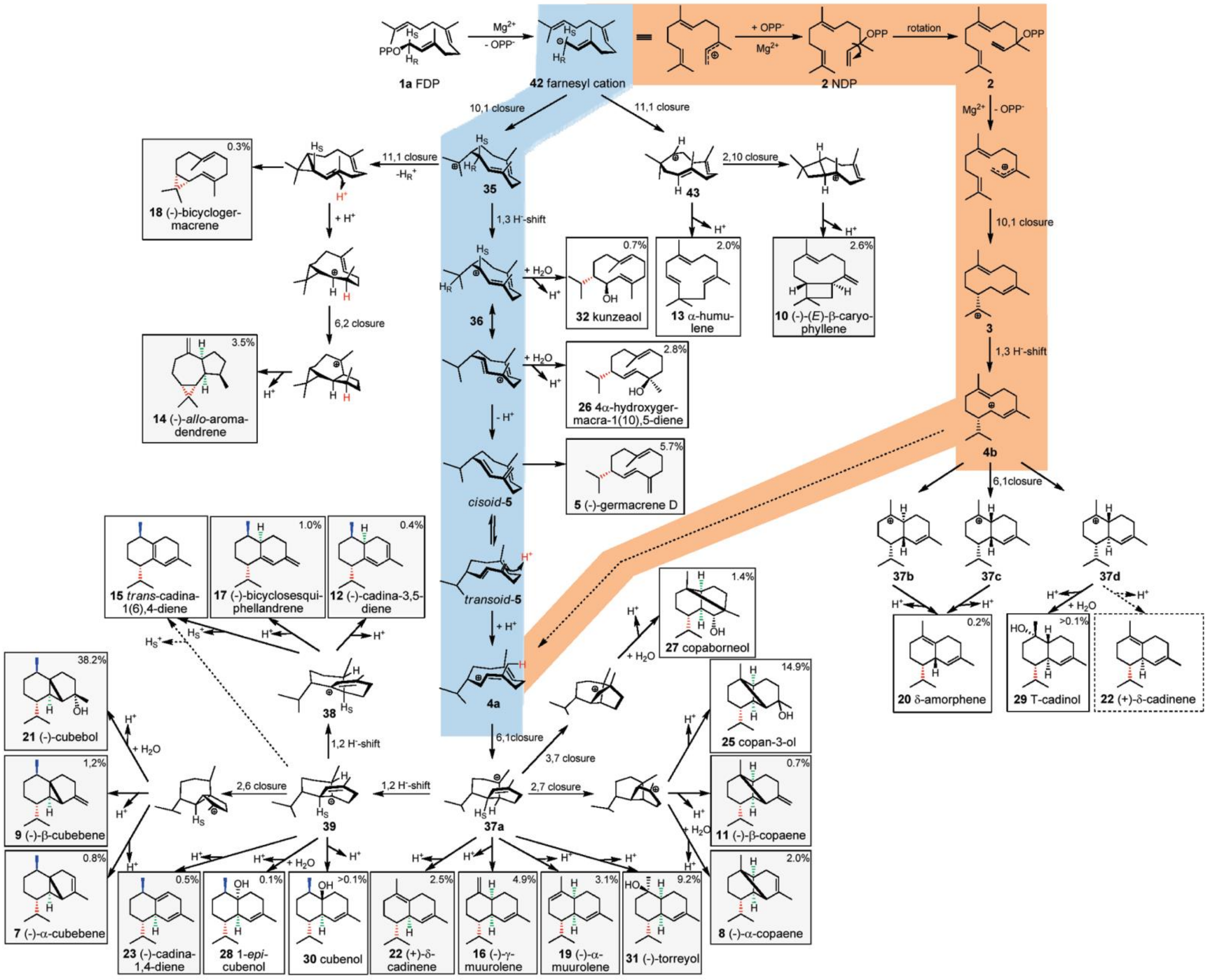

[a]Stereocenters with the same absolute configuration are marked in green, red, and blue. Reaction channels leading to **4a**,**b** by protonation of germacrene D (**5**) (light blue) or by the intermediacy of NDP (**2**) (orange) are highlighted. Absolute configuration determined (gray highlighted boxes).
